# Bacterial metabolism in the host and its association with virulence

**DOI:** 10.1080/21505594.2025.2459336

**Published:** 2025-01-31

**Authors:** Amrita Bhagwat, Tiyasa Haldar, Poonam Kanojiya, Sunil D. Saroj

**Affiliations:** Symbiosis School of Biological Sciences, Symbiosis International (Deemed University), Pune, India

**Keywords:** Intracellular pathogen, metabolism, immune-metabolites, metabolic-cross talk, commensal, competition

## Abstract

The host restricted pathogens are competently dependent on their respective host for nutritional requirements. The bacterial metabolic pathways are surprisingly varied and remarkably flexible that in turn help them to successfully overcome competition and colonise their host. The metabolic adaptation plays pivotal role in bacterial pathogenesis. The understanding of host-pathogen metabolic crosstalk needs to be prioritized to decipher host-pathogen interactions. The review focuses on various aspects of host pathogen interactions that majorly involves adaptation of bacterial metabolism to counteract immune mechanisms by rectifying metabolic cues that provides pathogen the idea of different anatomical sites and the local physiology of the host. The key set of metabolites that are recognized as centre of competition between host and its pathogens are also briefly discussed. The factors that control the timely expression of virulence of bacterial pathogens is poorly understood. The perspective presented herein will facilitate us with a broader view of molecular mechanisms that modulates the expression of virulence factors in bacterial pathogens. The knowledge of crosslinked metabolic pathways of bacteria and their host will serve to develop novel potential therapeutics.

## Introduction

In the bacterial system, the cellular activities like energy production and building blocks are fuelled by a central mechanism of metabolism. Though there are a lot of variations in growth pattern and niche occupied, the majority of basic metabolic tasks are common across organisms. For instance, all organisms have to scavenge nutrition through highly co-ordinated central metabolism. Further the information of the metabolic state is to be regulated in the cell so that there is uniform availability of nutrients and flow of energy to carry out vital processes [1]. The entire process of metabolism enters into a complex mode during the bacterial infection, where bacteria have to thrive on nutrients from other organisms. From a bacterial standpoint, mammalian host is a dynamic system that has a plethora of many perfectly adapted resident bacteria which are potential competitors of the invaders. These residents have developed efficient ways to process available nutrients as well as active mechanisms to protect their environment against competing bacterial species. In order to understand how bacterial metabolism influences infection, it is critical to take into account both the pathogens metabolic potential and the specific host environment in which the bacteria proliferate. The host environment serves more than just a nourishing source for microorganisms. Also, host cells manage their own metabolism on a continuous basis, using the nutrients that are available and eliminating waste [2]. Also, the host resident microbiome have constant check on the invading microorganisms. Therefore, inorder to establish successful infection the pathogen must encounter the constant pressure from host immune system as well as fight with commensals present at the particular niche. These characteristics that modulate the relationship between pathogen and its host forms the basis of any infection, be it mild or severe. An “arms race” is most commonly used description for interactive relationship of microbial diseases and human immunity. The final outcome of this race is determined by the interaction between the immune effectors that directly aim to destroy bacterial survival and the countermeasures put forward by pathogen to overcome these attacks [3]. The site of infection can be thought as a closed system wherein metabolic processes of two or more organisms are simultaneously operating. In such conditions, both microbes and host use metabolic cues to sense the state of infection and modify the environment for their own advantage. As a result, even a small change in this interconnected metabolic system, brings about a significant impact on the course of an infection. In case of intracellular pathogens this becomes even more true, where pathogens have to directly compete for nutrients like amino acids and carbon sources with the host cells in which they reside. Similarly, which results into the byproducts of bacterial fermentation are released into the host directly where they bring change in host’s metabolic state by triggering diverse reactions. Therefore, for a microbe to cause infection, it needs to adapt to a shifting metabolic environment and avoid being eliminated by the host immune system [4].

This virulence driven metabolic pathways are crucial to establish infections and are often focused on the competitive acquisition of a few critical essential nutrients [5]. Recently, it has been reported that a few sets of metabolites, especially amino acids like arginine, tryptophan and asparagine are very important for both host as well as bacteria, for which they compete and form a metabolic interface [2]. The review aims to re-evaluate the shared metabolic interfaces between the pathogen and its host from a metabolism centric point of view. Here, we discuss about how pathogens have adapted their physiology to actively exploit the metabolic substrates generated by a robust inflammatory response, how they specifically modulate host metabolism to dampen the immune response, and how sensing the metabolic state of the local environment provides important cues to the bacterium regarding both the anatomical site of infection and the immune status of the area. A pervasive theme of these studies is the dominant role of interlinked host and pathogen metabolism in determining the outcome of an infection [4]. The knowledge of these interlinked metabolic pathways can be idealistic targets for developing new antimicrobial treatments, since the invasive bacteria exploits various host pathways for causing pathogenicity. The pathogenic microbes quickly acquire resistance to current antibiotics, the new anti-infective strategies are aimed to prevent development of resistance rather than killing the microbes [6]. For these research needs to be channelised towards bacterial metabolism that plays role in virulence rather than just targeting the traditional virulence factors. Studies using mutations are frequently used to identify metabolic genes, although these genes are rarely studied further. Future research may concentrate on the metabolic genes of the bacteria that may be implicated in host-pathogen interactions. Antimicrobial treatments that target bacterial metabolism haven’t proven very effective up until now. The virulence factors of bacteria are being re-examined as potential targets for therapy [7]. The concept of targeting bacterial virulence traits or metabolism could be of key importance in designing the therapeutics, as these targets will not exert the survival pressure on bacteria, leading to resolving the issue of antimicrobial resistance [4,7]. To summarise, there has to be clear understanding of bacterial metabolism specific to infection of the host that will help in improving our knowledge of bacterial pathogenesis as well as host metabolism. This will also provide insight to develop new antimicrobial strategies targeting specific pathogens. In this review, we will examine the successful human pathogens like *Neisseria meningitidis, Streptococcus pyogenes, Listeria monocytogenes, S*. Typhimurium and *Mycobacterium tuberculosis*; their metabolic adaptability highlights the importance of sensing the metabolic environment of the host, adapting to changing nutrient availability and competing with the host for metabolites.

## Metabolic controls of host–pathogen interaction and alteration in bacterial metabolism according to changing host niche

Like all bacteria, intracellular bacteria are classified as heterotrophs, which means they rely on external carbon sources for energy generation and the production of building blocks for essential cellular components [8]. Host niches can provide up to 100 different carbon substrates along with various other essential elements like nitrogen, phosphorus, and sulphur. However, these environments also present challenges with limited concentrations of key divalent cations (Mg2+, Mn2+, Fe2+) [9]. The alimentary canal undergoes chemical composition variations across its length, and bacteria that live there utilise these cues as signposts. It has been observed that carbohydrate is one of the common cues provided. Certain pathogens, like invasive streptococci, can feed on sugar to obtaining glucose and α-glucan, which are abundant carbohydrates that help identify the oropharynx [10]. Bacterial catabolism of these substrates fuels cellular energy production through the generation of reducing electron donors like NADH and FADH_2_ and ATP. Furthermore, catabolic processes serve as a critical source of essential biosynthetic precursors for anabolic pathways. These precursors include key intermediates of central carbon metabolism such as glucose-6-phosphate, fructose-6-phosphate, phosphoenolpyruvate (PEP), and acetyl-CoA. Additionally, catabolism can generate propionyl-CoA, oxaloacetate, and α-ketoglutarate, which serve as the precursor for various cellular components [11]. Certain pathogens, like streptococci, reside on the skin tissue and plentiful amount of simple and complex carbohydrates are available on surface of cell tissues. On the contrary, intracellular pathogen like *Mycobacterium tuberculosis* rely on the limited available carbon sources. These are referred to as “low-carb” pathogen [10]. Most bacteria are able to obtain carbon from a variety of elements. The carbon sources can either be co-metabolize by bacteria or used as readily available carbon sources. Carbon catabolite repression (CCR) is a key mechanism in bacteria which allow them to choose the best fuel source for the growth [12].

### Neisseria meningitidis

The process of meningococci colonisation begins when the bacteria adhere to the human nasopharyngeal epithelial cell layer. Meningococci pass through the blood–brain barrier, adhere to the endothelial cell layer of brain vessels, cross the epithelial cell layer of the nasopharynx, enter the bloodstream, and eventually multiply in the CSF of the subarachnoid space. Additionally, they must evade the human immune system. During infection, meningococci come into contact with a host environment having distinct metabolic challenges concerning host immune effectors and nutrition availability. For the bacteria to colonize the human nasopharyngeal mucosa for an extended period of time, its metabolism must adapt to the hostile environment. It does this by using lactate, which is catabolized more quickly than glucose. Lactate, which is produced and excreted as a part of glucose fermentation in human cells. Lactate has previously been shown to stimulate metabolism and oxygen consumption in pathogenic *Neisseria* [13,14]. *Neisseria meningitidis* relies on carbon source utilization to evade complement-mediated immune responses, linking its metabolism to virulence. Disruption of the lctP gene, encoding lactate permease, impairs growth in cerebrospinal fluid and reduces resistance to complement killing. This effect might be even more pronounced during active meningitis, where lactate levels in CSF soar while glucose concentrations dwindle. Furthermore, it was found that utilization of lactate might have a link with LPS sialylation in the bacteria. The mutant’s virulence is restored in complement-deficient models, with attenuation tied to the sialic acid biosynthesis pathway [13]. This pathway generates a molecule that acts as a phosphate donor for regulatory systems governing gene expression during infection, potentially including genes involved in LPS sialylation [15]. The TCA cycle activity and, in turn, the intracellular pool of 2-oxoglutarate are influenced by the availability of various carbon sources. Genes related to the core metabolic pathway and nutrient transport were shown to have variable expression in human blood as observed in ex vivo transcriptome analysis. Genes responsible for haemoglobin receptor, transferrin binding protein, transporters of glucose and lactose, along with TCA cycle and glycolysis genes were found to be upregulated [16,17]. Moreover, the utilization of various extracellular carbon sources, including lactate, pyruvate, and acetate, contributes to the enhanced survival of *N. meningitidis* within macrophages by elevated production of capsular polysaccharides. Moreover, cysteine is the preferred sulphur source for *N. meningitidis* [18].

### Mycobacterium tuberculosis

Fatty acids, with long carbon chains, hold significantly more potential energy than glucose. Consequently, their complete oxidation through the process of beta-oxidation yields a greater number of ATP molecules per molecule of substrate. *M. tuberculosis* enters the respiratory tract through aerosols and is usually internalized by alveolar macrophages in the lung. The infected macrophages transport the pathogen to deeper tissue regions and draining lymph nodes, where these macrophages become part of the granuloma in which *M. tuberculosis* is able to persist in a latent, metabolically quiescent state for a long time [19]. Although it has been shown in different models that *M. tuberculosis* uses different carbon sources at different stages of the infection process, it is generally accepted that host lipids are the primary carbon source for *M. tuberculosis* in vivo [20]. Glucose maybe accessible to *M. tuberculosis* in the macrophage intracellular milieu, but it is not the primary carbon source. *M. tuberculosis*, exploit host fatty acids to enhance their survival, persistence, and virulence within the host. These fatty acids are critical during infection for energy production, immune evasion, and adaptation to stress. Fatty acids serve as an efficient carbon and energy source, especially during nutrient scarcity in granulomas. Mycobacteria metabolize these lipids via beta-oxidation and store them as triacylglycerols (TAGs) for future use. Lipid metabolism supports bacterial survival in oxygen-deprived environments, a hallmark of chronic infections. Mycobacteria utilize host fatty acids to synthesize unique cell wall lipids like phthiocerol dimycocerosate (PDIM), which masks pathogen-associated molecular patterns (PAMPs) [21]. Additionally, lipid uptake influences foam cell formation, providing a protective niche [22]. This dual role of host fatty acids as an energy reservoir and a tool for immune manipulation highlights their importance in *M. tuberculosis* pathogenesis. Targeting lipid metabolism pathways in *M. tuberculosis* or disrupting foam cell formation could provide innovative therapeutic strategies for combating tuberculosis [21]. In addition, different pathogenic bacteria have a finely tuned system for managing nitrogen, a vital element for building molecules. This system ensures the bacteria can balance creating its own nitrogenous compounds with taking them in from its surroundings, especially during shortage [23]. *M. tuberculosis* mainly uses a specific pathway called glutamine synthetase-glutamate oxoglutarate aminotransferase (GOGAT) to incorporate nitrogen from ammonium, its preferred source. Ammonia, a readily available but potentially toxic nitrogen source, can fuel rapid bacterial replication [24]. *M. tuberculosis*, prioritize amino acids as their nitrogen source. Amino acids not only provide essential building blocks for bacterial growth but also serve as precursors for virulence factors [20].

### Streptococcus pyogenes

Pathogenic streptococci is responsible for a variety of diseases, from minor ones like dental caries to serious ones like pneumonia and sepsis. These bacteria grow on the host’s extracellular surfaces, such as dental biofilms or the tissues of the nasopharynx in humans. They are set established here to either get nutrition directly from the host during self-feeding or to obtain it from the extracellular surfaces of the host tissues. Streptococcal genome has co-evolved with the changes in the human diet so as to utilise the available carbohydrates. This is particularly the case for the EII permeases of the phosphoenolpyruvate:sugar phosphotransferase system (PTS), a multi-enzyme pathway of carbohydrate uptake found in most bacteria. Furthermore, *S. pyogenes* appears to have a flexible CCR system that enables bacteria to control which carbohydrate to use for energy catabolism by detecting intracellular nutrient levels. As a result, in the invasive strain of *S. pyogenes*, the PTS and CCR system regulate the production of virulence genes including streptolysin S[25].

### Listeria monocytogenes

Ammonia is a nitrogen source, can fuel rapid bacterial multiplication and can induce virulence genes in certain pathogens [26]. L-glutamine induces virulence in *L. monocytogenes*. *L. monocytogenes* is a food-borne pathogen that enters the intestinal lumen initially. Following their passage through the intestinal epithelium, these bacteria are taken up by phagocytic cells, including granulocytes, dendritic cells, and macrophages. *L. monocytogenes* senses the abundance of branched chain amino acids (BCAAs) to determine the hosts cytosolic environment inorder to adapt and regulate its virulence during intracellular infection. The isoleucine-binding transcriptional regulator CodY, which interacts with the master virulence regulator PrfA, is a key component in this regulation system. Through direct binding, CodY, a worldwide transcriptional regulator, detects the amounts of BCAAs, especially isoleucine. CodY experiences an allosteric alteration that allows it to either activate or repress target genes when BCAA levels are elevated. CodY fine-tunes the bacterial response to the host environment by controlling genes linked to metabolism, stress adaptability, and virulence. In *L. monocytogenes*, PrfA is the main virulence regulator, regulating the expression of important virulence genes, such as those necessary for intracellular survival and phagosome escape.

### *Salmonella* Typhimurium

Another illustration of nutrient sensing during adaptation to the host environment and virulence, the resulting virulence gene expression profile is crucial for the bacteria’s intracellular growth and survival [27]. *S*. Typhimurium may survive the stomach’s acidic pH and enter the small intestine after consuming contaminated food or water. There, it adheres itself to and invades the epithelial cells of the small intestine. *S*. Typhimurium can cause a more serious systemic infection if it penetrates the intestinal epithelium, spreads to the mesenteric lymph nodes, and then enters the liver and spleen. *S*. Typhimurium can detect the intestinal environment through the sensing of other chemical signals that are specific to this site, such as bile salts. Complex strategies have been developed by *Salmonella* to penetrate and colonise host cells that are elaborated by Jorge E. in his manuscript [28]. A diverse range of carbohydrates and gluconeogenic substrates may be assimilated and processed by *S*. Typhimurium. Thus, the outcome of an infection with *S*. Typhimurium depends on the presence and availability of certain carbon sources inside host tissues. Once the gastrointestinal lumen has been colonised, *S*. Typhimurium invades the epithelial mucosa by injecting effector proteins into host cells through the type III secretion system 1 (T3SS–1), which is encoded by Salmonella pathogenicity island 1 (SPI-1). In Salmonella, certain carbon sources like glucose, pyruvate, or fatty acids affect key metabolic processes including glycolysis and the TCA cycle. Metabolites produced by these pathways have the ability to either directly or indirectly function as signalling molecules to control the expression of T3SS–1. The availability of carbon sources modulates T3SS–1 expression in *Salmonella Typhi* through metabolic sensing and transcriptional regulators like CRP-cAMP, Cra, and HilA. These pathways ensure that T3SS–1 is expressed when conditions favour invasion or survival, integrating metabolic and environmental cues for virulence control. A systemic infection with *S*. Typhimurium causes the TCA cycle to be suppressed and glycolytic activities to be upregulated, which causes host cell inflammatory responses and metabolic reprogramming. Instead of depending on oxidative phosphorylation, *S*. Typhimurium infected tissues favour aerobic glycolysis as a means of energy production. The detailed mechanism of metabolic shift during systemic *Salmonella* infection are discussed in detail by Wang et. al that can be referred [29]. In the gut milieu, which is rich in bile, simple carbohydrates like lactose and glucose are crucial energy sources. To effectively metabolise these sugars, *S*. Typhimurium modifies its metabolic pathways, especially when bile salts are blocking alternative carbon sources. *S*. Typhimurium may break down peptides produced from the host or scavenge amino acids from host proteins. The bacteria ensures a consistent supply of nitrogen and carbon sources for energy generation by upregulating amino acid transporters and enzymes involved in amino acid catabolism in response to bile salts.

## Metallophores as key players in bacterial pathogenicity

Metallophores are very small, high-affinity chelators released by microbes to sequester vital metals from the host environment. The ability of pathogenic bacteria to overcome host-imposed nutritional immunity- a defence mechanism in which the host restricts microbial access to metals including iron, zinc, manganese, and copper – requires the presence of metallophores [30]. The function of metallophores in the pathogenicity of *Neisseria meningitidis*, *Streptococcus pyogenes*, *Listeria monocytogenes*, *S. Typhimurium*, and *Mycobacterium tuberculosis* are discussed.

*N*. *meningitidis* produces siderophores that sequester iron from host transferrin and lactoferrin for its survival and virulence. TbpA and TbpB proteins are known to facilitate the uptake of iron from transferrin. LbpA and LbpB systems enable the pathogen to thrive in the cerebrospinal fluid by targeting lactoferirin bound iron, where iron availability is tightly regulated [31]. *S. pyogenes* produces streptobactin that helps it in scavenging iron. It also employs alternative metallophore systems (AdcABC system) to access manganese and zinc, critical for combating oxidative stress and DNA repair during infection [32]. Similarly, *L. monocytogenes* uses metallophore system like HupDGC system that captures haem-bound iron. Fhu system allows *L. monocytogenes* to survive within phagosomes and contribute to its intracellular lifestyle during infection. In *S. Typhimurium* there are multiple siderophores, like salmochelin, aerobactin and enterobactin that aids the bacteria to overcome host defences. Salmochelin, evades host lipocalin-2 and neutralizes host siderophores. Manganese and zinc uptake systems are essential for oxidative stress resistance and survival within macrophages. These metallophore systems are indispensable for *S*. Typhimurium’s survival in iron-deprived environments such as the intestinal lumen and intracellular compartments [33]. The mycobactin family of siderophores lies at the core of *Mycobacterium tuberculo*sis’s special iron acquisition mechanism. Iron is reportedly extracted from host ferritin and transferrin by carboxymycobactin and mycobactin [34]. Furthermore, *M. tuberculosis* depends on manganese and zinc transporters to preserve enzymatic processes necessary for intracellular survival and to combat oxidative stress. Metallophores are pivotal in the pathogenic strategies of diverse bacterial pathogens. By hijacking host metal pools, these molecules enable bacteria to bypass nutritional immunity, sustain metabolic activity, and establish infection. Understanding the molecular mechanisms governing metallophore synthesis and uptake offers potential targets for developing antimicrobial therapies aimed at disrupting metal homoeostasis in pathogens [35].

## Host immunometabolites regulating bacterial pathogenicity

**Figure 1.**
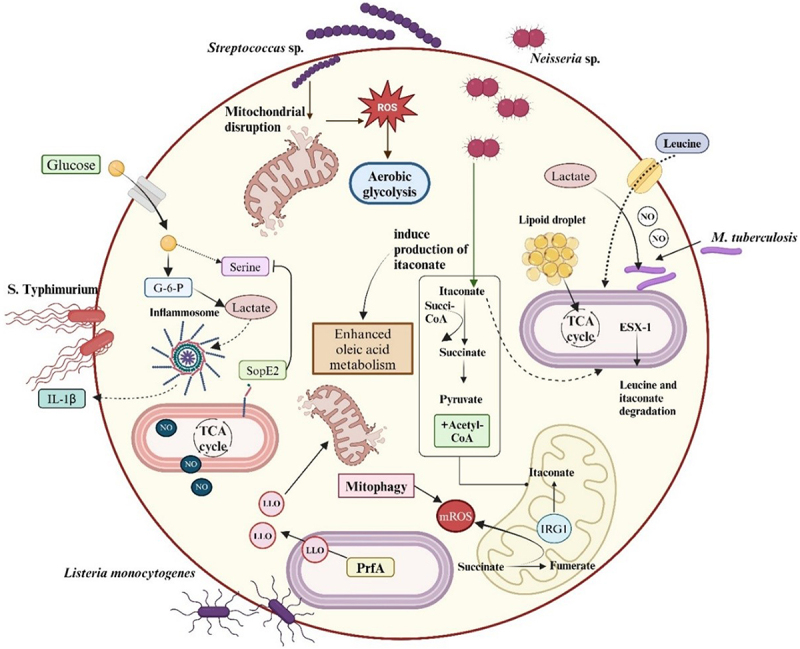
Demonstrates the general physiological function of immunometabolites in adaptation of pathogens. *N. meningitidis* can potentially alter the production of crucial immunometabolites such as itaconate, promoting the disease progression. *L. monocytogenes* employs prfA to regulate major virulence factors such as production of listeriolysin O by exploiting the host metabolites. *mycobacterium tuberculosis* demonstrates metabolic flexibility within macrophages, utilizing the ESX-1 secretion system for survival. *streptococcus* can cause mitochondrial disruption in macrophages resulting in a metabolic shift towards glycolysis.

The traditional understanding of host–pathogen interactions primarily focused on the recognition and eradication of invading pathogens by the immune defence mechanism. Recent studies discovered that host immune metabolites play a critical role in shaping infection outcomes [36] (Figure 1). The host immune cells trigger a sophisticated and well-established metabolic remodelling after recognizing the pathogen bacteria. Macrophages significantly shift from a basal, relatively inactive metabolic state to a highly activated state upon encountering bacterial pathogens. This immunometabolic reprogramming plays a crucial role in prioritising the product of biosynthetic and energetic precursors that are important to shape the robust immune defence mechanism to eliminate the infection. Metabolic reprogramming is also attributed to regulating the expression of innate and adaptive immune cells for producing chemokines, cytokines, interferons, and antimicrobial procedures [37]. Studies suggest that bacterial pathogens can detect and utilise immunometabolism and enhance their chances of survival, causing successful infection [38]. Table 1 provides information about the role of immune metabolites and the metabolic interactions between pathogens and the host.

**Table 1.** Role of host metabolites in virulence of pathogens.

Name of bacteria	Key metabolites	Virulence	References
*Neisseria meningitidis*	Polyamines	Increase the synthesis of capsular polysaccharide Increase the survival in macrophages	[39]
	Lactate, pyruvate, and acetate	Increase the intracellular survival in macrophages	[39]
	Itaconate	Regulate the oleic acid metabolism and ceramide biosynthesis	[40]
*Streptococcus pyogenes*	Phosphoenolpyruvate	Production of virulence genes including streptolysin S	[25]
	Glutathione	Innate immune evasion	[41]
*Streptococcus pneumoniae*	Glucose	Enhance glycolytic enzyme activity and favour interconversion of pyruvate to lactate dehydrogenase	[42]
*Mycobacterium tuberculosis*	Cholesterol esters and TAG	Survival in the host cells	[21]
*Listeria monocytogenes*	Glucose-6-phosphate and glycerol	Increase production of listeriolysin positive regulatory protein (PrfA)	[43]
S. Typhimurium	Malate, pyruvate, lactate, and succinate	Trigger the same encouraging better survival of the bacteria in host cells	[44]

### Recognition of pathogens and metabolic remodelling of macrophages

The macrophage has emerged as a paradigmatic model cell for investigations within the escalating field of immunometabolism. These cells switch between pro-inflammatory and anti-inflammatory states, each with a unique metabolic profile [45]. Exposure to Toll-like Receptor (TLR) agonists, such as bacterial lipoproteins, peptidoglycan, DNA, or lipopolysaccharide, triggers a metabolic shift towards aerobic glycolysis as a result of receptor identification activating signal transduction cascades [46]. Because of these metabolic preferences macrophages rapidly generate ATP to sustain their potent effector functions which include the production of inflammatory cytokines and microbicidal activities. Conversely, exposure to anti-inflammatory signals activates a switch to mitochondrial respiration, supporting tissue repair [47]. This metabolic change creates specific molecules that help macrophages carry out their different functions during infection and healing. The remodelling causes increased citrate and cis-aconitate synthesis, controlled by two mechanisms i.e. the type I interferon pathway represses isocitrate dehydrogenase I leading to citrate accumulation and lipid biosynthesis in activated immune cells [38,48]. Secondly, the activation of a signalling pathway by nuclear factor NF-κB drives the production of nitric oxide (NO) which directly inhibits both isocitrate dehydrogenase I and cis-aconitase. The reliance on mitochondrial respiration supports the reparative and tissue remodelling functions characteristic of anti-inflammatory macrophages [49]. Upon immune activation, myeloid cells crank up the production of an enzyme called immune-responsive gene1 (IRG1) which converts cis-aconitate to itaconate by utilizing the leftover enzymes from the disrupted TCA cycle [50]. Itaconate acts as a metabolic regulator and plays a crucial role against microbial infection. Delivered by a specific protein guanosine triphosphatase RAB32 to acidic compartments within phagosomes, itaconate disrupts bacterial metabolism by blocking isocitrate lyase to hinder the use of certain fuel sources like acetate and propionate [51]. In addition, the conversion of citrate to itaconate by IRG1 causes succinate build-up. Therefore, HIF-1α is released from its prolyl hydroxylase (PHD)-mediated repression by succinate and mitochondrial ROS which helps to eradicate the bacterial infection [52]. However, some pathogens have developed a way to break down itaconate, potentially as a means to counter the metabolic disruptions caused by immune cells. The detailed molecular mechanism of itaconate modulating virulence in bacterial pathogens is mentioned previously by Rosenberg et al. [38].

### Immunometabolites supporting bacterial growth

During the infections, the invading pathogen retorts and exploits host-secreted metabolites as major nutrient sources. Interestingly, it was observed that bacteria can influence the host cells to release itaconate by modifying their membrane which creates a positive feedback loop promoting chronic infections. Therefore, the exchange of molecules promotes a refined communication channel that allows both the host and pathogen to flourish by utilizing itaconate as a nutrient source. Itaconate is one of the crucial immunometabolites that plays a fascinating role in metabolic warfare. Itaconate possesses antimicrobial properties therefore, at high concentrations, it can be toxic to both host and pathogen [53]. In the airway of some bronchiectasis patients, *Neisseria* sp. is found to trigger inflammatory responses which in turn promotes airway immune remodelling. During infection, production of coenzyme Q10 was increased which acts as the predominant fuel source of activated immune cells and pyrimidine ribonucleotides [40]. Upon infection, *Neisseria* cause alteration in itaconate production and oleic acid metabolism which can potentially damage the host cells. Perhaps, the most concerning is the upregulation of ceramide biosynthesis which leads to severe lung damage. Iron and lactate are two immunometabolites critical for growth of *N. meningitidis*. These resources highlight the intricate interplay between host immune responses and bacterial strategies for survival. Lactate serves as an alternative carbon source for *N. meningitidis*, supporting its metabolic flexibility under nutrient-limiting conditions. During streptococcal infection, the systematic metabolic adaptability of carbohydrate metabolism provides functional efficiency to the immune cells [54]. Throughout symptomatic colonization, *S. s pyogenes* disturbs the mitochondrial function in macrophages. This disruption causes a decline in ATP production which in turn enhances oxidative stress. As a response, macrophages undergo a metabolic shift from efficient mitochondrial respiration to aerobic glycolysis. This transition involves increased glucose uptake, enhanced glycolytic enzyme activity and favoured interconversion of pyruvate to lactate dehydrogenase [42]. Glucose is the key immunometabolite that supports the growth of *S. pyogenes*. Free fatty acids, lipids, and glucose are released by host cells during inflammation, and *S. pyogenes* uses these nutrients for growth, membrane synthesis, and production of energy. During infection, L. monocytogenes depends on immunometabolites produced by the host. Glucose, glycerol, and amino acids – which supply energy and the building blocks for cellular functions – are important metabolites that sustain its growth [55]. The bacteria scavenges iron from host proteins, which is frequently sequestered during inflammation. Fatty acids, lipids, and polyamines obtained from the host are essential for metabolic adaptability and memsbrane production. Its intracellular survival is also maintained by nucleotides from lysed host cells and metabolites produced during immunological reactions, such as lactate and succinate. Such strategies allow *L. monocytogenes* to remain virulent while adapting to an array of environments [43]. Upon interaction with macrophages, *S. Typhimurium* forms Salmonella-containing vacuole (SCV), characterized by nutrient scarcity necessitating metabolic flexibility. Acute Salmonella infection models comprise a robust pro-inflammatory response with macrophages relying heavily on glucose metabolism [44]. In contrast, chronic infections exhibit a passive immune response with macrophages switching to fatty-acid oxidation. This metabolic remodelling generates an opportunity for *S. Typhimurium* to flourish inside the host cells by exploiting the host fatty acids which benefits them to persist longer through glyoxylate shunt. Interestingly, *S. Typhimurium* not only utilizes the host immunometabolites as a source of energy but also senses them as crucial signalling molecules that potentially activate different virulence factors. In addition, host-derived pyruvate, lactate, and succinate can trigger the same, encouraging better survival of the bacteria in host cells. While pyruvate and lactate act through membrane signalling, succinate uptake is essential for virulence induction by *S. Typhimurium*. On the other hand, macrophages develop an intimidating environment for pathogenic bacteria by restraining the availability of crucial nutrients like different ions. *Mycobacterium tuberculosis* exhibits remarkable metabolic plasticity within macrophages, exploiting the early secreted antigenic target 6 (ESX-1) secretion system for survival [56].

## Metabolic pathways can be co-opted as a virulence strategy

Bacterial metabolites can function as means of communication, influencing various physiological processes. Many microbes, including commensals and pathogens, use diffusible signal molecules to coordinate multicellular behaviour – a mechanism known as “quorum sensing.” Quorum sensing is known to regulate important traits of bacteria such as competence, adherence, virulence gene expression, and stress response at the population level. Pathogens or other bacteria may use some metabolites produced by other bacteria/hosts as a food source or colonisation aid. These metabolites can function as potential cues to alter the expression of genes involved in virulence of the pathogen. Hence, we can say that pathogens can exploit various metabolic pathways to their advantage as a virulence tactic – in this case as signalling molecules [57]. Indole is an example of one such bacteria derived metabolite which plays an important role as a signalling molecule in microbiota-host interactions. Tryptophan serves as the precusor for indole synthesis and microbes exhibit a crucial role in tryptophan metabolism [58]. The expression of the tna operon, which codes for tryptophanase, increases in response to elevated extracellular tryptophan concentrations. The environmental tryptophan uptake is regulated by TnaA and TnaB permeases which are transcribed as an operon. The extracellular concentrations of indole are greatly affected by pH, temperature and presence of carbon sources. Indole molecules have the capacity to activate genes linked to succinate or act on regulatory proteins that have a variety of biological roles, including spore or biofilm production, virulence, plasmid stability, and drug resistance [59]. In *L. monocytogenes* it was demonstrated that indole-rich conditioned medium and synthetic indole significantly decreased the expression of certain virulent characteristics like cell aggregation, exopolysaccharide synthesis, biofilm formation as well as motility. Indole plays role in attenuation of *Salmonella* Typhimurium (*Salmonella*) virulence by decreasing the invasion to J744A.1 macrophages. This decrease in invasion was attributed to presence of indole that resulted in downregulation of various *Salmonella* Pathogenicity Island-1 (SPI-1) genes. Additionally, it was also observed that indole synergistically amplified the inhibitory effect of different short chain fatty acid cocktailed together against SPI-1 gene expression [60].

The hoemostasis between metabolites secreted by the host and its microbiota are primarily governed by the complex and dynamic microbial community that resides in the human gut. Numerous QS signal syntheses rely on environmental metabolites. Biosynthesis of AI-2 forms an essential part of central bacterial metabolism since it is a part of activated methyl cycle, which is one of the aspects of methionine metabolism. AI-2 is produced from S-adenosylmethionine (SAM) via a three-step enzymatic process. Methionine is recycled using homocysteine. Some bacteria only do this, absorbing AI-2 and reintroducing it into the body when necessary. There is reduction in bacterial fitness when LuxS is disrupted, which in turn affects the pathogenicity by reducing the biofilm formation. Various bacteria use AI-2 as a signalling molecule in modulating their population size as well as use it to activate a specific regulatory response via quorum density (QS). Pathogenicity will be affected greatly if the QS regulon encompasses virulence factors. There would be major phenotypic changes observed on disruption of either AI-2 or LuxS, since both have potential roles in bacterial metabolism as well as quorum sensing. Looking at LuxS role in bacterial pathogenesis, the review aims to focus on LuxS-dependent phenotypes of pathogens that inhabit certain niches within the host [61].

*N*. *meningitidis* occasionally disseminates into the bloodstream causing bacteraemia and later spreads into the central nervous system. The bacteria exhibits AI-2 activity that is dependent on *lux*S homologue. Not much of difference was observed in the transcriptional profile of *N. meningitidis* luxS mutant and its wildtype. The *lux*S mutant was able to recover and was capable of adhering to human epithelial cells. Through these experiments it can be concluded that *lux*S in *N. meningitidis* does have a role in production of signalling molecules but might have an important role in its metabolism [62,63]. On the contrary, LuxS in *S. pyogenes* has been reported to govern virulence traits. *S. pyogenes* while causing infections of soft tissues often secrete Streptolysin (causes haemolysis) and a secreted cysteine protease i.e SpeB, that are regulated by LuxS. Siller et al. [64], have reported that in *lux*S mutant there are reduced levels of SpeB with enhanced bacterial invasion rates as compared to wildtype [64]. A luxS deficient mutant of *L. monocytogenes* produced a denser biofilm as compared to its wildtype. Supplementation with exogenous AI-2 failed to restore the phenotype in the mutant, bringing to the conclusion that luxS gene is responsible for repression of attachment and development of a biofilm. The decline of biofilm in mutant can be caused by the depletion of nutrients and accumulation of toxic compounds which induce bacterial detachment in *L. monocytogenes* [65]. In Salmonella the synthesis of AI-2 and transcription of pfs are found to be closely associated. This can result into increase in SAH detoxification since it enhances Pfs level that in turn boost SRH levels and LuxS activity. Therefore, high levels of AI-2 will play a role in metabolism rather than quorum sensing. The internalisation of AI-2 is encouraged by one of the gene present in Lsr operon, which depicts its role in metabolism. These findings suggest that *lux*S mutant has an impact on regulation of genes of Lsr ABC transporter which is employed to transport AI-2, also will affect its utilisation as a metabolite. On comparing this *lux*S mutant with the parental strain it showed altered *met*E expression, wherein metE is involved in de novo synthesis of methionine [66]. Pathogenic bacteria are not the only organisms that can produce AI-2, but also a variety of probiotic bacteria and commensals, including strains of *Lactobacillus* and *Bi-fidobacterium*, have *lux*S homologues and are capable of producing AI-2.

## Metabolites from commensals and host microflora in regulating bacterial pathogenesis

The human body is home to over 500 microbial species. The host microbiota, a complex assemblage of microorganisms, plays a vital role in host pathogen interaction, characterised by a mutual exchange of resources and metabolites [67]. Bacteriocins, protein toxins produced by the microbiota, offer a natural defence mechanism against unwanted competitors. These targeted weapons eliminate specific bacteria while leaving beneficial microbes unharmed. Bacteriocins exploit unique vulnerabilities in their targets. Some disrupt the bacterial cell wall. Others infiltrate the pathogen and interfere with its genetic machinery, halting growth. Certain bacteriocins can even inhibit protein synthesis within target bacteria. On the contrary, bacteriocins from many pathogenic bacteria can alter the host microbiota [68].

Gut bacteria produce short-chain fatty acids (SCFAs) such as butyric, propionic, and valeric acids showing bactericidal activity against *Salmonella* by disrupting their membranes and lowering internal acidity. Furthermore, *S. Typhimurium* actively manipulates the gut environment by promoting inflammation, thereby suppressing the growth of butyrate-producing bacteria like *Clostridia* spp. This strategy eliminates competition for resources while simultaneously allowing *S. Typhimurium* to exploit the inflammatory byproducts. Interestingly, sub-inhibitory SCFA concentrations significantly reduced *Salmonella* motility and, in some strains, biofilm formation. Enzyme action in host-microbiome causes fatty acids to be liberated from host lipids, resulting in free fatty acids (FFAs) [69]. When unsaturated FFAs pass the cell wall or outer membrane of bacteria, they most likely attach directly to the transporters of the electron transport chain or intrude into the inner membrane, causing the electron carriers to separate or be displaced from the membrane. These processes indicate that FFAs can impact bacterial energy metabolism by disrupting the electron transport chain [70]. The persistence of *M. tuberculosis* within a host has proved to be linked with beta-oxidation and glyoxylate metabolic processes. In mice, fatty acids are the major source of carbon for *M. tuberculosis*. This includes the usage of beta-oxidation, which converts fatty acids with an even number of carbon atoms into acetyl-coenzyme A (CoA), while fatty acids with an odd number of carbon atoms are converted into acetyl-CoA and propionyl-CoA. Thus, lipid metabolism allows mycobacteria to survive in a host.

Host-derived vitamins possess a complex interplay between gut bacteria, vitamins, and pathogens. While vitamins are essential for human health, their production and impact on pathogens present a fascinating duality [71]. The human gut houses bacterial species like *E. coli*, lactic acid bacteria, and bacteroides, which synthesize vitamin K2 (VK2). Microbiota derived VK2 are found to be absorbed by the colon [72]. However, conditions like small-intestinal bacterial overgrowth (SIBO) can influence the VK2 levels. SIBO does not influence the bacterial production of VK2 but it can potentially trigger intestinal damage [73]. Interestingly, even some potential pathogens like *L. monocytogens* can synthesize VK2 playing a critical role in pathogen survival and virulence [74]. In *L. monocytogenes* VK2 plays a critical role in aerobic respiration as menaquinone is a key component of the electron transport chain. Stritzker et al., showed that mutant deficient in VK2 production force the bacteria to undergo anaerobic metabolism significantly impairing the survival and virulence [75]. On the other hand, in *L. monocytogenes* σB acts as the major transcriptional regulator of different stress response genes. In the σB regulon, four-gene transcription units were found to be involved in menaquinone (VK2), folic acid, and potentially riboflavin biosynthesis [76]. Also, vitamin B2 are found to regulate host immune response which in turn control the pathogenicity in *L. monocytogenes* through VB2-dependent NADPH oxidase 2 (Nox2) activation [77].

### A metabolic pathogenic strategy that disadvantages the microflora

While pathogenic bacteria are adept at causing harm, their virulence mechanisms can have unintended consequences for the natural, resident microflora. These metabolic pathways, designed to give pathogens an edge in the host environment, can create disadvantages for the surrounding microbial community. One key example is competition for resources. Pathogens often encode for efficient nutrient acquisition systems or synthesize unique molecules that target the host resources. This aggressive behaviour can deplete essential nutrients or oxygen, leaving less for the resident microflora. For instance, some pathogens produce siderophores, high-affinity iron chelators, that outcompete resident bacteria for this vital element. Another disadvantage imposed by pathogens is the production of antimicrobials [33,78]. Many pathogens synthesize bacteriocins or toxins specifically targeting competing bacteria. These molecules can disrupt the growth or survival of resident species, altering the delicate balance of the microflora [79]. Furthermore, some pathogens manipulate the host immune response in ways that indirectly harm the microflora. By triggering excessive inflammation or disrupting immune tolerance, pathogens can create an environment hostile to the resident bacteria. Disruption of the microflora by pathogens can have cascading effects on the host’s health. A weakened microflora can lead to increased susceptibility to further infections, impaired digestion, and even contribute to inflammatory bowel diseases [80]. Unlike the nutrient-rich gut, the respiratory tract offers inadequate pickings for bacteria, especially for opportunistic pathogens. These bacteria struggle to survive due to limited access to a key molecule NAD+ [81].

### Metabolic interdependence and competition are key factors in bacterial pathogenesis

The availability of primary metabolites like amino acids is crucial for bacterial growth. Beyond fuelling pathogens, metabolite availability shapes the host immune response. Different immune cells have varying metabolic needs. This creates competition for resources, influencing both pathogen growth and the nature of the immune response [80]. One such example is the competition for tryptophan, which the host depletes through the action of IFNγ-induced IDO. This pressure likely explains why many pathogens, despite losing the ability to synthesize other amino acids, retain pathways for tryptophan synthesis either complete or partial [82]. Asparagine plays a critical role apart from bacterial physiology. It serves as a vital nitrogen source during infection. Interestingly, asparagine is also essential for host lymphocyte replication. Salmonella *Typhimurium* exemplifies this competition [83]. *S. Typhimurium* manipulates the host adaptive immune response to persist. Recent research reveals that *S. Typhimurium* secretes an L-asparaginase enzyme that depletes asparagine, thereby hindering lymphocyte activation and protective immunity [84]. *M. tuberculosis* also relies on host asparagine for various purposes. It utilizes asparagine for nitrogen acquisition and to withstand the acidic environment within the phagosome of macrophages.

### A metabolic switch can turn a commensal into a pathogen

**Figure 2.**
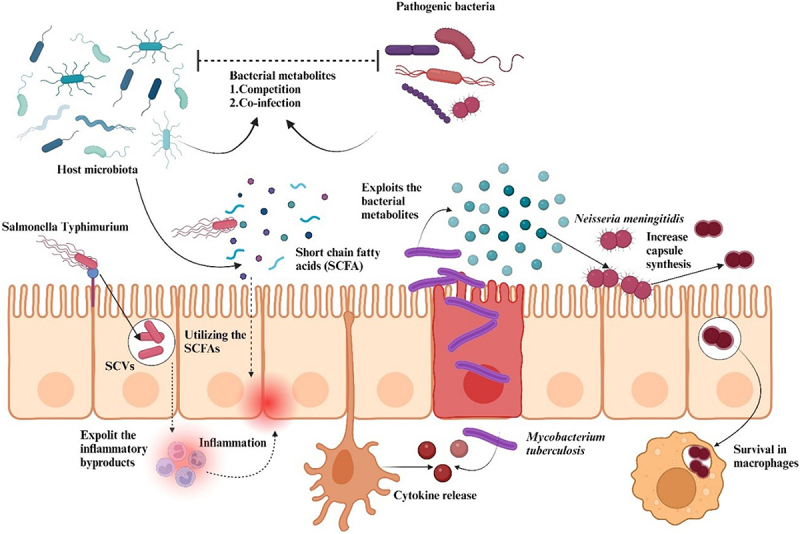
The host-microbiota plays a vital role in host–pathogen interaction, characterized by mutual exchange of resources and metabolites. Pathogenic bacteria can exploit the specific metabolic intermediates in two different ways, either by competing with the neighbouring bacteria or by co-utilizing the same metabolites.

Imbalance of microorganisms in the gut can lead to various conditions in the human host. Some recent studies also demonstrated that overgrowth of a variety of *Lactobacillus* spp. such as, *L. fermentum*, *L. coryniformis*, *L. acidophilus*, *L. rhamnosus*, are linked with pleuro-pulmonary infections. Similarly, other commensal bacteria like *Enterococcus* and *Bifidobacterium* can turn into opportunistic pathogens causing infections [85]. The intestinal epithelium faces a constant immunological challenge i.e. discriminating between commensal microbes and enteric pathogens. A study focused on uridine utilization in bacteria found that harmful bacteria and pathogens tend to use uridine as fuel which triggers an immune response in the gut lining, generating chemicals that kill these harmful microbes. Conversely, good gut bacteria lack the enzymes needed to utilize uridine, allowing them to coexist peacefully. Nucleoside hydrolase (NH) enzyme plays a critical role in allowing pathobionts to break down uridine. Enteric pathogens and pathobionts possess NH enzymes enabling them to catabolize uridine for growth [86]. This uridine catabolic ability is absent in commensal gut bacteria. By eliminating NH activity, the pathobionts were essentially disarmed. They could no longer utilize uridine, and consequently, the dual oxidase (Duox) system, responsible for generating toxic ROS, remained inactive. Interestingly, inactivation of the NH enzyme in *P. aeruginosa* resulted in a significant decrease in the production of AHL-type quorum-sensing molecules which can potentially contribute to the symptomatic transition of the bacteria from an asymptomatic state [87]. Figure 2 demonstrates the role of microbiota in host–pathogen interaction.

## Conclusion

The complex interplay between metabolism and virulence requires a critical understanding as to how both of them function individually and parallelly. It becomes challenging to mark the boundaries between the two since they share various links in the bacteria. At times bacterial virulence factor production drains the metabolic pathways, leaving impaired pathogenicity and reducing fitness of bacteria. An example to this is, unlike AI-2 other bacterial signalling molecules (Quorum sensing signalling molecules-QSSMs) cannot enter the feedback loop of metabolism because they are produced by dedicated synthases, with the goal of interacting with cognate regulators, e.g. alkyl quinolones (AQs) and N-acyl homoserine lactones (AHLs). Due to QS the signalling molecules accumulate, but in due course the metabolic pathways related to the generation of the substrates from which they arise are drained in the process of producing QSSMs. The feeder metabolic pathways involve the synthesis of amino acids and fatty acids, and when the substrates from these metabolic pathways are drained, virulence is negatively impacted by a fitness cost. The impact is significant enough to support the existence of bacteria that do not produce QSSMs but respond to other bacteria’s signalling molecules, thus conserving their energy. Therefore, these non-producing bacteria benefit in a way that they have a reserve of their metabolites if encountered with adverse conditions and can spread infection efficiently. Likewise in nutrients scarce conditions, persisters i.e metabolically inert cells maintain themselves by reducing their metabolic rates and slow growing. They can be primarily found in depths of biofilms or during antimicrobial encounters. Certain virulence factors can supply nutrients that confer an edge in terms of fitness. For instance, Pseudomonas aeruginosa’s surface-tethered aminopeptidase autotransporter, AaaA, can produce arginine locally that the bacteria can metabolise. This may be a survival tactic in low-oxygen conditions like a biofilm microcolony.

Therefore, it becomes crucial to decipher the underlying mechanisms of central metabolism that affect the generation of virulence factors and pathogenesis in bacteria. From the perspective of a bacterium, in order to successfully colonise the pathogen needs to adjust to a unique set of challenges in order to minimise competition and obtain access to resources. During colonisation the bacteria uses the metabolic cues to alter their gene expression and metabolism, which inturn optimises the bacterial growth and helps it survive in a particular niche. These metabolic cues can be provided either by other bacteria in its vicinity or by the host itself. As soon as the host senses an infection various immune responses targeting the bacterial metabolic process are elicited, and to counter this, bacteria alter or twist its metabolic pathway. As mentioned in this review, host immune evasion can be achieved through various unique changes in metabolic network like- by loss of catabolic repression, induction of alternative pathways or acquiring new genes etc. The fact that both host and pathogen cells share nutrient pools also means that there are lots of possibilities for each to manipulate the local chemical environment, compete directly for metabolic substrates like amino acids, and detect the other’s physiological status.

Hence understanding the metabolic flexibility and constraints of bacterial infections under specific host conditions will allow us to develop rational therapeutics that would target vulnerabilities in each vital metabolic network of a pathogen. Unfortunately, due to lack of technology and thorough methods to investigate metabolism during infection are still in progress our understanding of these critical adaptations and metabolic shifts has lagged behind as compared to bacterial pathogenesis. Although requirements for certain metabolic enzymes may be deduced using traditional genetic techniques for analysing bacterial pathogenesis, each of these enzymes can participate in several pathways and frequently carry out reversible processes. Hence, the dissection of metabolic pathways necessitates the combined use of more complex biochemical techniques and genetics. Advancement in mass spectrometry can accurately characterise the microbial metabolism during growth of a bacteria and determine its metabolic status at the site of infection. However, estimating the concentrations of these metabolites from a particular organelle or tissue can be challenging as their distribution varies greatly. Gaining a greater comprehension of these points where host and pathogen metabolism converge might lead to the identification of basic processes underlying metabolic predispositions to illness as well as novel therapeutic approaches. Ultimately, new therapeutic intervention options will arise from the identification of the metabolic pathways linked to susceptibility to infection. It is becoming more and more common to find agents that may alter host metabolism. Modulating the interlinked metabolic system between microorganism and host could be a valuable adjunctive therapy, especially for antibiotic-resistant pathogens. This is especially true for chronic pathogens that continue to balance host immunity. The current intersection of technological progress and interest in the metabolic basis of disease manifestation, promises to give numerous opportunities to test this method, even though the final benefit of metabolic modulators in infectious disease has to be demonstrated [36,78,88–90].

## Data Availability

No dataset is generated in this article.
